# Proceedings of the Louisville Medical Club

**Published:** 1855-08

**Authors:** John Bartlett

**Affiliations:** Secretary


### Art. II.—PROCEEDINGS OF THE LOUISVILLE MEDICAL CLUB.

REPORTED BY JOHN BARTLETT, M. D., SECRETARY.

The Louisville Medical Club met at the house of Dr. Yandell on the evening of the 9th of August.

Vice-President Knight occupied the chair, and the following gentlemen were present: Drs. Gross, Marshall, Rogers, Richardson, Thomson, Bemiss, Yandell, Raphael, D. W. Yandell, Knight, Bartlett, and Caldwell.

Dr. Rogers introduced Dr. Sexton, of Indiana, and Dr. Yandell introduced Dr. Hubner, of Louisiana.

The observations of members on ulcers of the *frenum linguæ* in hooping cough being called for,

Dr. Gross reported that since the last meeting of the Club he had examined eighteen cases of hooping cough of different ages, and in different stages of the disease, without discovering any ulcers under the tongue in but two instances. One of these was a boy, aged two years, who had had the disease for ten days. The cough was frequent and violent. An ulcer, small and superficial, with diphtheritic edges, existed under the tongue at the frenum. The other case occurred in the person of a girl three and a half years old, who had been sick four weeks. She had a large diphtheritic ulcer under the tongue, superficial but very sore; parts around inflamed and red. A small ulcer, of a similar character, existed on the under surface of the inferior lip.

Dr. Rogers reported having seen twenty-seven cases of hooping cough, and found an ulcer under the tongue in a single case. In this subject the disease had existed for four weeks. Dr. R. re marked that the observation of his partner, Dr. Pirtle, had been very different, he having seen the frenum ulcerated in a considerable proportion of his cases.

Dr. Rogers was able to furnish the Club with the history of sixteen other examples communicated to him by his friend, Dr. Brewer, a practitioner of this city. Of these, eleven were found to have an ulcer on the frenum linguæ. The ages of the patients, and the duration of the hooping cough, were respectively as follows: three years, duration two weeks; two years, duration ten days (ulcer just forming); five years, duration two weeks; two years, duration three weeks; three years, duration two weeks; nine years, duration ten days; two years, duration four weeks; four years, duration one week (ulcer just forming); two years, duration four weeks; four years, duration two weeks; four years, duration six weeks.

Dr. Knight had examined thirteen cases and seen but one ulcer.

Dr. Yandell had examined but one case—in that there was no ulcer.

Dr. Caldwell had seen five or six cases, and detected an ulcer in only one. There were other ulcers in the mouth of this patient, and he could not say that the ulcer upon the frenum differed in its origin from these.

Dr. Thomson said that sore mouth prevailed to some extent in his practice just now—that he had observed several cases of hooping cough in which sore mouth existed, but he had not looked for the ulcer under discussion.

Dr. D. W. Yandell had treated six cases of hooping cough. In three there were diphtheritic ulcers in other parts of the mouth, but none beneath the tongue.

Dr. Barlett reported seventeen cases, and three ulcerations. The ages of the patients and duration of the disease were as follows: Six years, declining stage, no ulcer; six years, incipient stage, no ulcer; five years, two weeks, no ulcer; two and a half years, six weeks, no ulcer; eight years, declining stage, no ulcer; five years, five weeks, ulcer; two and a half years, second attack, four weeks, no ulcer; two years, three weeks, no ulcer; three years, three weeks, no ulcer; four years, three weeks, ulcer; same case at five weeks, no ulcer; three and a half years, four weeks, no ulcer; two years, four weeks, no ulcer; two and a half years, five weeks, no ulcer; seven months, five weeks, no ulcer; twelve years, four weeks, no ulcer; five years, four weeks, an ulcer; two years, four weeks, no ulcer.

Dr. Bartlett remarked that the most of these cases had been examined by him but once, others had been watched from the commencement.

Dr. Richardson had seen but one case of hooping cough. In this there was distinct ulceration of the frenum. Dr. R. suggested that the investigation of this subject be continued for further report, to which Dr. Gross added that he thought it desirable that members in their future observations should note the ages of their patients, the duration of the disease, the character of the ulcer, and the existence of sores in other parts of the mouth.

Dr. Yandell made the following remarks on

### COUP DE SOLEIL.

Under the term *coup de soleil*, or sun stroke, two affections are included of opposite characters. One is of the nature syncope or nervous exhaustion; the other is cerebral apoplexy, induced by intense heat. Both are peculiarly casualties of large cities.

In 1853, according to the report of the City Inspector, two hundred and sixty deaths in the city of New York were produced by sun stroke, exclusive of cases designated "congestion of the brain," and "the effects of cold water." The number of deaths from this cause was much greater during the summer following, the heat of which was intense and protracted beyond anything experienced for many years. In the course of that summer many cases of sun stroke were announced in Louisville.

As the name implies, the attack of sun stroke is sudden and overwhelming. The subjects, usually laborers, are seized with pain in the head, sense of oppression in the epigastrium, occasionally nausea and vomiting, prostration, vertigo, dimness of vision, and insensibility. From this condition the patient is sometimes speedily restored, as from fainting, but in more severe cases he passes, in a short time, into a state of coma. The skin is not cool, as might be expected in a case of exhaustion, but hot; according to Dr. Dowler the temperature is sometimes as high, in such cases, as 112° F. The pupils are generally dilated in this stage, but are occasionally contracted; pulse hurried and feeble, or full and slow; respiration laborious.

The case pursuing a fatal course, symptoms of collapse come on. The respiration becomes stertorous, accompanied with sighing and moaning; the general surface grows cool while the head remains hot; the sphincters become relaxed, the extremities cold, the pupils, generally, contracted; and the patient dies suddenly, or may linger for several hours.

Dr. Dowler makes a distinction between "solar exhaustion" and "solar asphyxia," the former being, according to his view, "a mere fainting," the patient having a pale, cool skin, while in the latter the skin is burning hot, the face flushed, the body insensible to impressions, the patient comatose. Solar exhaustion is easily remedied by proper treatment; solar asphyxia is generally fatal.

Of sixty cases of coup de soleil treated by Dr. Swift in the New York Hospital, in 1853, forty-four were insensible at the time of admission, and sixteen were either stupid or sensible. The pupils were dilated in thirty cases, contracted in nineteen, and in eleven natural. In twenty-four the body was hot, in fourteen warm or natural, and cool in twelve; the temperature of the head being elevated in thirty-one of the cases, warm in eleven, and cool in eighteen. The pulse was uniformly accelerated, varying from one hundred to one hundred and sixty. The average duration of Dr. Swift's fatal cases was about four hours. Of the sixty cases fifty occurred in males, and a large proportion of the subjects were foreigners, many of whom had but recently landed in the city. About one-half of the cases that occur in New York, according to Dr. Swift, are fatal. From 1845 to 1853, inclusive, one hundred and fifty cases were admitted into the New York Hospital, of which seventy-eight were fatal. In 1853, of sixty-seven cases thirty-three terminated in death.

Dr. Swift attaches much importance to the state of the pupils in cases of sun stroke. He found the pupils contracted in twenty out of thirty-three fatal cases, moderately dilated in seven, and markedly so in six; while in the cases resulting favorably, the pupils were dilated in nineteen, and nearly natural in fifteen. No case under his observation recovered in which the pupils were contracted. Dr. Swift remarked, also, that only two patients recovered in whom there was "sighing, moaning respiration."

The post-mortem appearances were discovered by Dr. Swift to be "opposite to those found in congestion of the brain, or apoplexy produced by insolation," and he examined the brains of all the patients in whose cases he suspected cerebral lesions. In such cases depletion is uniformly followed by death. Dr. Swift never saw a patient recover after he had been bled.

Dr. Condie holds that depletion is required in cases attended by hot skin and head, injected eyes, contracted pupils, small, quick, corded pulse, delirium, and restlessness. If not relieved by prompt and active treatment, he found that patients with these symptoms speedily became comatose, and died as in acute meningitis.

Dr. Swift states that the lungs are engorged with blood in the fatal cases.

The treatment in sun stroke is now, almost universally, stimulant. Depletion has been everywhere abandoned. Dr. Swift placed his patients in a hot bath, rendered more stimulating by mustard or capsicum, or had mustard applied to their bodies and extremities; gave stimulating enemas of comp. tinct. aloes, or spts. terebinth.; applied ice to the head when it was hot, and administered brandy, tinct. capsicum, and carbonate of ammonia, pro re nata.

Cases of coup de soleil have not been frequent in Louisville, but during the intensely hot weather, last summer, a number of laborers were stricken down and died in this condition. Lewis, the colored servant at the University, aged about fifty years, was prostrated while mowing the grass in the college yard. He was seen by Prof. Gross and myself, and recovered, rather slowly, under the use of stimulants. He remained for several weeks feeble, and was unable to labor in the sun.

Dr. Marshall asked if Dr. Yandell considered those cases simulating sun stroke but occurring after exertion in heated rooms, identical with coup de soleil? A man at the rolling-mill dropped down suddenly with insensibility; pretty active and strong pulse, flushed face, head hot, injected eyes and hot skin. He had just eaten his dinner and taken some whisky. He had not over-exerted himself. He was bled till he vomited, and his bowels were opened by an injection. He died in five or six hours.

He saw another case: A cook, who fell with about the same symptoms. He used injections, and applied cups and cold water to the head. She recovered.

Dr. Yandell thought these cases of exhaustion. He believed they required stimulants.

Dr. Gross remarked that exhaustion might even be present in apoplexy. In many cases, especially if the patient be a weak man, he should not at first be bled; stimulants might be required, or the patient should at least be kept quiet. Much mischief had been done by indiscriminate bleeding in apoplexy.

Dr. Richardson asked if the effect of sun stroke be occasionally limited to the spinal cord? He was treating a patient who was seized while breaking rock on a turnpike with stiffness and pain in the back of the neck, and a loss of sensibility in the superior extremities.

Dr. Yandell thought the effect of the stroke might be so limited.

Dr. Hubner had seen in Louisiana a number of cases. If delirium is present for more than twenty-four hours, it is impossible to save the cases. The pulse is usually full, and the pupils dilated.

Dr. Knight mentioned that he had seen a case of sun stroke a few weeks since. The subject was working at a brick-kiln. His pupils were dilated. The treatment was brandy, mustard to the surface, and cold applications to the head. Patient was insensible for ten hours, and after recovery complained of severe pain in the head, for which wet cups were applied. Slight hemiplegia remained for several weeks after recovery.

Dr. Caldwell saw a case in the summer of 1854. The man, a colored laborer, fell down insensible, with shivering, a full pulse, and dilated pupils. He recovered in half an hour under the use of brandy, and cold water to his head.

Dr. Caldwell mentioned another case which presented, at the outset, the phenomena of sun stroke but terminated in phthisis. A negro woman, while performing labor in the house on a hot day, last summer, fell down insensible. Dr. C. saw her in half an hour after the attack, and found her with a full pulse and pupils dilated. He had cold water thrown upon her head, and administered brandy and ammonia, under which treatment she became sensible in five or six hours, but fell into a typhoid state, in which she remained for several days. She then became troubled with cough, and exhibited in a short time all the symptoms of tubercular consumption, with which she died.

Dr. Bemiss treated a case of sun stroke last summer. The City Engineer, in the month of July, was prostrated in the street, but recovered in a few hours under a stimulant treatment, with cold water to the head, and strict rest. He treated a second case which promised also to terminate favorably, but the patient, an Irishman, having recovered his consciousness and the use of his limbs, attempted to walk home, and on the way fell asphyxiated and died.

Dr. D. W. Yandell exhibited Mr. Langston Parker's Apparatus for Mercurial Fumigation in syphilitic affections. Dr. Y. remarked that, owing to his having been comparatively unsuccessful in the first experiments he made with this mode of treatment, he had addressed a letter to Mr. Parker requesting a detailed description of his apparatus. Mr. Parker was kind enough to reply with a degree of minuteness which had enabled him to construct an apparatus similar in all essential respects to that used by the Birmingham surgeon. Dr. Y. had been unable to obtain the description of lamps mentioned by Mr. P., but had supplied their place with those of a form suggested to him by Professor J. Lawrence Smith. With those he could, with great uniformity, vaporize any of the preparations of mercury used in this method without raising the temperature beyond what was easily borne by patients.

The first person whom Dr. Y. submitted to mercurial fumigations with the proper apparatus, was a gentleman of intemperate habits, who had been the subject of secondary syphilis for nine months. During two-thirds of this time he had been under the care of one medical man of this city, and during the remaining three months he was under the care of another. His first medical attendant had given him the bichloride of mercury and the usual adjuvants. His second had used the protoiodide of mercury. Neither had done more than to keep the disease at bay. Under neither did any of the symptoms disappear. When Dr. Y. saw him in January the disease was well developed, both on the skin and tongue, in the throat and mucous membrane of the nose. His hair was falling out. At the end of three weeks, during which period he had taken twelve baths. the sores on the mucous surfaces and the discolorations of the skin had disappeared, and his hair had ceased to fall out. The patient used nothing internally but the compound decoction of guaiacum, prepared as recommended by Mr. Parker, after the Edinburg Pharmacopoia. The black oxide and the bisulphuret were the forms of mercury vaporized. They were used at alternate baths.

Of seven cases treated by this method, Dr. Y. had met with but one that did not yield with the most satisfactory promptitude. This occurred in the person of a gentleman who had gone through a long course of mercury in the hands of a physician of this place. His disease partook of the characters both of secondary and tertiary syphilis. The latter troubles did not materially improve till the patient had undergone fumigation for the space of three months. The secondary accidents disappeared at the end of a few weeks.

Another case which Dr. Y. wished to relate to the Club occurred in the person of a commercial man of this place, who, while on a visit to the Northern cities contracted a primary sore on the prepuce. He consulted a respectable German practitioner ten or fifteen days after the accident, and was subjected to severe cauterization of the chancre, and took internally a mercurial. This treatment was persisted in for two or three weeks. In the meantime the glands in the groin commenced enlarging and the sore on the genitals showed no disposition to heal. Mercurial frictions over the enlarged glands were ordered and the mercury continued internally. At this juncture the patient applied to Dr. Yandell. His condition was as follows: In the left inguinal region the glands were greatly enlarged and indurated. At the inferior and internal edge of the tumefaction there was considerable tenderness on pressure. The sore on the prepuce was about the size of a pea and presented all the characteristics of an indurated chancre. There was some constitutional disturbance and a very depraved state of the primæ viæ. Remedies were directed for the latter conditions; the chancre was dressed with opium cerate; the glands in the groin were painted with tr. iodine, and pressure applied, and the patient enjoined to observe perfect rest. This he did not do. The tumefaction over the point that has been described as being tender on pressure continued to increase and became exceedingly painful. It finally suppurated and was opened—the abscess was carefully washed out—pressure reapplied and healing occurred by the first intention. The chancre cicatrized but left in its stead an induration equal to its own original size. The swelling and hardness of the inguinal glands continued unabated. The patient quit his bed and returned to his business. Mercurial frictions over the groin alternated with tr. iodine liberally applied to the surface, and constant and well adjusted pressure by means of the roller, were continued for two weeks. No change occurring at the expiration of this time in either the site of the chancre or the glandular enlargement, other remedies were suspended and the patient was placed in the vapor bath. Internally he used the decoction of guaiacum. After the third bath a perceptible diminution was manifest both in the inguinal swelling and the seat of the preputial sore. In six weeks all traces of both had entirely disappeared. The patient's gums had become tumefied, red, and somewhat tender, but there was no ptyalism. The intervals between the baths were now gradually lengthened and the patient discharged at the end of another month cured.

Inquiry being made as to the preparations of mercury Dr. Y. is in the habit of using, he remarked that he used those recommended by Mr. Parker. Indeed, he said, his treatment was in every respect that of the British surgeon. After the perfection of the apparatus required by this method Dr. Y. had found most difficulty in obtaining mercurial preparations in a state of purity. Whatever form is used it is indispensable that it be pure. On being asked by Dr. Gross what proportion of relapses had occurred, Dr. Y. observed that a sufficient length of time had hardly yet elapsed to speak with any positiveness as to the permanence of the cures related, but that as far as his observation extended he thought the per centage of relapses less than where the disease was treated by Ricord's method. In but one of the cases in which the fumigations had recived a satisfactory trial had there been a return of the disease, and this was clearly traceable to over-indulgence in wine.

Dr. Y. promised a full report of his cases at some future meeting of the Club. For the present he wished merely to call the attention of the members to this mode of treatment, and to express his satisfaction with the results that it had, thus far, yielded in his hands.

Dr. Knight was requested to give at the next meeting the results of his observations in regard to the use of Chloroform in labor—especially as to its effects upon uterine contractions, and its influence, if any, in producing asphyxia of the child.

Dr. Thomson was also requested to furnish the Club, at the same time, with the results of his experience in the use of the same remedy in surgery.

The Club then adjourned to meet on the first Tuesday in September at the house of Dr. Bemiss.

